# DUSTrack: Semi-automated point tracking in ultrasound videos

**DOI:** 10.1038/s41598-026-42795-3

**Published:** 2026-04-24

**Authors:** Praneeth Namburi, Roger Pallarès-López, Jessica Rosendorf, Duarte Folgado, Brian W. Anthony

**Affiliations:** 1 Institute for Medical Engineering and Science, MIT, Cambridge, MA 02139 USA; 2 MIT.nano Immersion Lab, MIT, Cambridge, MA 02139 USA; 3 Department of Mechanical Engineering, MIT, Cambridge, MA 02139 USA; 4 Fraunhofer Portugal AICOS, Porto, 4200-135 Portugal; 5 Comprehensive Health Research Center (CHRC), Porto, 4200-135 Portugal

**Keywords:** Computational biology and bioinformatics, Engineering, Health care, Mathematics and computing, Medical research

## Abstract

Ultrasound technology enables safe, non-invasive imaging of dynamic tissue behavior, making it a valuable tool in medicine, biomechanics, and sports science. However, accurately tracking tissue motion in B-mode ultrasound remains challenging due to speckle noise, low edge contrast, and out-of-plane movement. These challenges complicate the task of tracking anatomical landmarks over time, which is essential for quantifying tissue dynamics in many clinical and research applications. This manuscript introduces DUSTrack (Deep learning and optical flow-based toolkit for UltraSound Tracking), a semi-automated framework for tracking arbitrary points in B-mode ultrasound videos. We combine deep learning with optical flow to deliver high-quality and robust tracking across diverse anatomical structures and motion patterns. The toolkit includes a graphical user interface that streamlines the generation of high-quality training data and supports iterative model refinement. It also implements a novel optical-flow-based filtering technique that reduces high-frequency frame-to-frame noise while preserving rapid tissue motion. Semi-automated tracking with DUSTrack demonstrates superior accuracy compared to contemporary zero-shot point trackers and performs on par with specialized methods. This establishes its potential as a general tool for clinical and biomechanical research—one that can generate accurate training data for developing foundation models in ultrasound point tracking. We demonstrate DUSTrack’s versatility through three use cases: cardiac wall motion tracking in echocardiograms, muscle deformation analysis during reaching tasks, and fascicle tracking during ankle plantarflexion. As an open-source solution, DUSTrack offers a powerful, flexible framework for point tracking to quantify tissue motion from ultrasound videos. DUSTrack is available at https://github.com/praneethnamburi/DUSTrack.

**Supplementary Information:**

The online version contains supplementary material available at 10.1038/s41598-026-42795-3.

## Introduction

Tracking tissue motion—including that of skeletal muscles and organs—has clinical, engineering, and scientific applications. For example, tracking the heart wall or liver motion^1^ can inform medical diagnoses, surgical planning, and disease progression monitoring. Tracking skeletal tissues can aid in identifying injury risk^2^ and developing effective physical therapy and rehabilitation protocols^3^. In sports science, tracking muscle and connective tissue dynamics can help optimize athletic performance and training methods^4^. Beyond practical applications, measuring tissue motion can reveal fundamental insights into the biomechanics and neuromuscular control of human movement.

Ultrasound imaging offers unique advantages for studying tissue motion in vivo. Unlike magnetic resonance imaging (MRI), which restricts participants to confined spaces, ultrasound allows nearly unrestricted movement during data collection. While fluoroscopy and dynamic computed tomography (CT) can image moving body structures^5–8^, their radiation exposure makes them unsuitable for non-critical applications. Other methods like sonomicrometry^9^ and magnetomicrometry^10^ can capture tissue motions at high spatiotemporal resolutions but require invasive procedures. Ultrasound imaging is safe, non-invasive^11^, and provides excellent spatial (micrometer-scale) and temporal (~300 Hz) resolutions, making it suitable for capturing dynamic tissue behaviors—from slow respiratory movements^12^ to rapid cardiac or muscular activities during running^13^.

Point tracking—the ability to track arbitrary points in videos—provides a foundational approach to analyzing the rich information captured in B-mode ultrasound data. Rather than building custom algorithms to measure specific features like fascicle length, muscle boundaries, or arterial diameter, a robust point-tracking system can serve as a versatile solution. By accurately following specific points of interest over time, we can derive higher-order features such as muscle area, localized torsion, and pennation angle. Therefore, point tracking sets the foundation for creating reliable, generalized tracking-based measurement frameworks.

Automated tracking in ultrasound B-mode videos remains challenging due to inherent limitations such as speckle noise^14,15^, out-of-plane motion^16^, and poorly defined tissue boundaries^17^. Current tracking approaches are prone to two types of inaccuracies: “drift” and “jitter.” Drift refers to low-frequency tracking errors that accumulate over time, while jitter describes high-frequency frame-to-frame noise in position estimates. Traditional computer vision methods, especially those relying on optical flow algorithms, struggle with drift-based inaccuracies during extended tracking periods^18^. While recent deep learning approaches, especially convolutional neural network (CNN)-based trackers, often outperform traditional methods, they frequently produce jittery outputs that compromise the reliability of derived measurements like tracker velocity and acceleration^19^. While combining these approaches could potentially address their respective limitations, developing an effective integration strategy remains challenging. Recent attempts at reducing both drift and jitter have been application specific. For example, UltraTimTrack^20^ excels at fascicle tracking by addressing both drift and jitter, but it is not designed for the broader task of tracking arbitrary points in B-mode ultrasound videos. To address drift and jitter in the generalized context of point tracking, here we introduce DUSTrack (a **D**eep learning and optical flow based toolkit for **U**ltra**S**ound **Track**ing).

DUSTrack is a novel, semi-automated, open-source toolkit for tracking arbitrary points in ultrasound videos. It employs deep learning frameworks to process spatial features within individual video frames, eliminating drift, and refines temporal trajectories using a novel optical-flow-based filter to minimize jitter.

An ideal point-tracking tool should excel in three key areas: generalization (across anatomical locations and imaging conditions), accuracy (precise point localization with minimal drift and jitter), and automation (minimal manual intervention). In developing DUSTrack, we prioritized generalization and accuracy to create a crucial stepping stone toward this ideal solution. The toolkit, supported by a graphical user interface, facilitates the generation of high-quality point trajectories, which can be used as ground truth data for training fully automated point-tracking pipelines.

## Methods

**Fig. 1:**
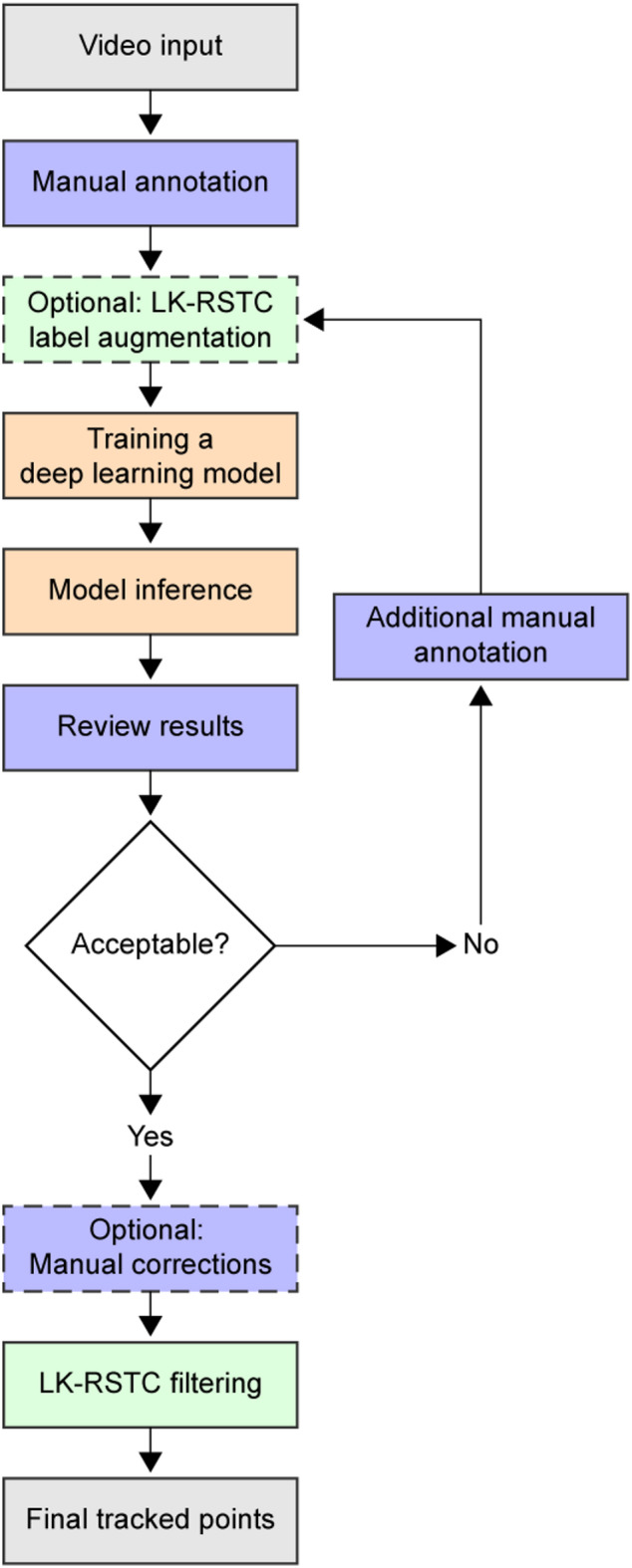
DUSTrack workflow. The process starts with video input and manual annotation of key frames. These annotations can optionally be augmented using the LK-RSTC optical flow algorithm (dashed box). The annotations are used to train a deep learning model, such as the ResNet-50. After model inference, results undergo review. If the results are unsatisfactory, users can make additional manual annotations to refine the model through iterative training. Once results meet quality standards, optional final manual corrections can be made before applying LK-RSTC filtering to produce the final tracked points. Purple indicates manual steps, light orange indicates deep learning steps, and light green indicates optical flow steps.

DUSTrack integrates deep learning and optical flow techniques to deliver accurate point tracking in ultrasound videos. The method (Fig. 1) consists of three main components: (1) a user-friendly application to generate reliable training data for deep learning algorithms, (2) deep learning-based tracking to eliminate drift, and (3) optical flow refinement to reduce jitter in the tracking results.

The first step generates labels for fine-tuning existing deep learning models. Traditional annotation methods for natural videos typically select several frames that are highly dissimilar and require annotators to manually identify matching landmarks across disconnected frames^21^. Although effective in natural video contexts, this approach becomes impractical for ultrasound videos due to the inherent complexity of ultrasound imaging, including speckle noise^14,15^, nonlinear tissue deformation^17^, and out-of-plane motion^16^. These challenges make it difficult for human annotators to reliably identify corresponding anatomical points across temporally separated frames, leading to potential inaccuracies and inconsistent annotations.

In response, we adopt an alternative annotation strategy informed by common practices in ultrasound interpretation, where annotators naturally track points through sequential frames. To operationalize this intuitive approach, we developed a graphical user interface (UI) that streamlines the annotation process, allowing users to follow points through sequential frames. The UI automatically generates intermediate tracking estimates between manually annotated frames using the Lucas-Kanade^22^ optical flow algorithm with reverse sigmoid tracking correction (LK-RSTC)^18^, reducing manual effort while augmenting the training dataset for deep learning models.

The LK-RSTC algorithm is a key component of DUSTrack. We briefly summarize this previously published algorithm^18^ here. Given a tracked point’s labels in two video frames (start and end), the algorithm estimates the point’s position in the intermediate frames. It tracks the point using the established LK algorithm^22^ in both forward (start to end frame) and reverse (end to start frame) directions. At each intermediate frame, it weighs the estimated locations from both tracking paths using a sigmoid function.

Built-in verification and correction tools enable users to review and refine these estimates, producing high-quality annotations suitable for training deep learning models. This step typically produces a few hundred labeled frames, with approximately 25 frames labeled manually (in the span of about 500 sequential frames) and the rest are augmented using the LK-RSTC algorithm. The augmentation step is optional (see Results). The result of the first step is a human-readable (.json) file containing the positions of annotated points that can be easily converted to any desired format. The second step involves training a deep learning model using annotated frames. While DUSTrack’s annotations can be used with various deep learning frameworks, the toolkit is designed to work with DeepLabCut (DLC)^23,24^, for example, by being able to read and write DLC-compatible annotation files. Through DLC, users can fine-tune state-of-the-art neural networks, including ResNet^25^, MobileNet^26^, and EfficientNet^27^. A key advantage of these models is that they process frames independently without using temporal information, which prevents the accumulation of tracking errors (drift) commonly seen in sequential tracking methods. After training, our interface enables users to visualize DLC tracking results, make additional annotations, and iteratively refine the model. When DLC estimates are unsatisfactory, users can override them either manually or by using the LK-RSTC algorithm for improved tracking accuracy. Finally, the optical flow refinement step employs the LK-RSTC algorithm once again to reduce jitter in manually-refined DLC estimates. While a simple low-pass filter could achieve this, utilizing the LK-RSTC algorithm better preserves fast tissue movements while minimizing jitter (see Results).

The optical flow refinement step uses a “transposed” sliding window filter. Unlike traditional sliding window filters, which estimate the signal at each point by averaging neighboring points temporally, our transposed variation averages multiple estimates derived from different overlapping windows encompassing the same frame. This approach mitigates jitter by capitalizing on the short-duration accuracy of LK-based estimations while maintaining motion characteristics.

For example, consider a 50 Hz ultrasound video analyzed using a sliding window of 0.6 s (30 frames). Within each window, a “tracklet” is computed, producing intermediate estimates across 28 frames using LK-RSTC. As the window moves forward, additional overlapping tracklets are computed, and the final position of any given point is the average across all tracklet-derived estimates encompassing that point. This averaging process exploits LK’s established low short-term drift and jitter characteristics^18^, addressing limitations found when directly interpolating between sparse deep learning predictions.

Within DUSTrack, Lucas-Kanade optical flow serves three interconnected yet distinct purposes: generating reliable reference annotations to enhance initial human labels, augmenting training datasets for deep learning models, and filtering the deep learning model outputs. Collectively, these integrated methods are supported by prior research validating these techniques in complex video tracking contexts^18,23,24^.

**Implementation details.** ResNet-50 networks were fine-tuned using default DLC parameters in versions 2.3.8 and 2.3.9, with the TensorFlow backend for the results reported in this manuscript. The LK-RSTC algorithm’s key parameter—the sliding window size—was set to 0.5 s. High-pass and low-pass filters were implemented using the pysampled Python package with its default parameters. Additional parameters for comparative filters are detailed in Fig. S1. For complete parameter specifications and reproducibility information, consult the DUSTrack codebase and its accompanying documentation.

### User interface

**Fig. 2:**
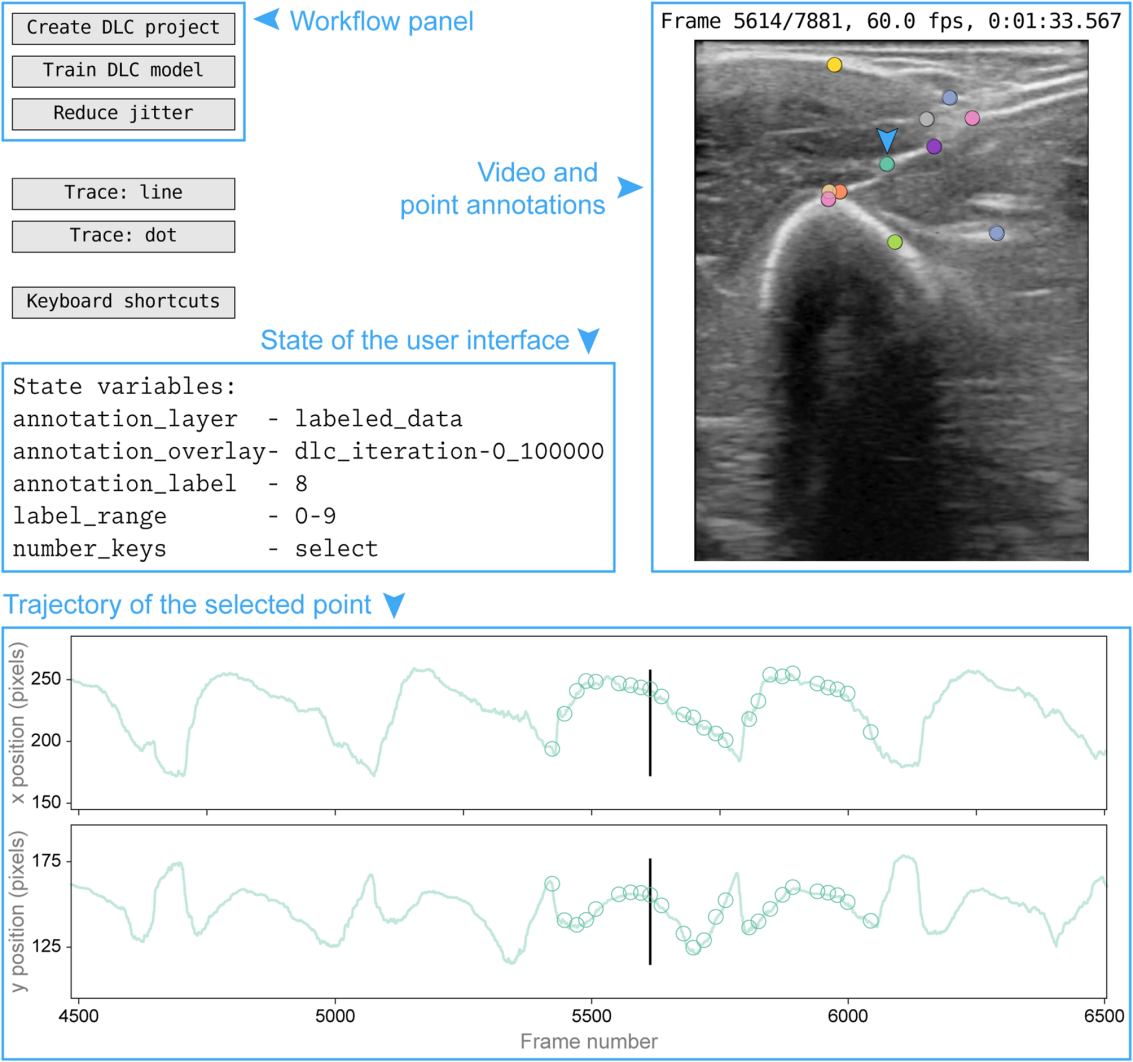
DUSTrack’s graphical user interface. The workflow panel (top left) provides controls for creating DLC projects, training models, and reducing jitter. The video and point annotations panel (right) shows the ultrasound video frame with current point annotations. The selected point is marked here with an arrow. The interface state panel (middle left) displays active variables, including the current annotation layer and selected point. At the bottom is a trajectory panel with plots showing the x and y coordinates of the selected point over time. The interface can display two annotation “layers” simultaneously—in this example, the “labeled_data” layer appears as open circles in the trajectory panel, while the “dlc_iteration-0_100000” layer appears as a translucent continuous trace. Blue text, arrows, and boxes have been added to highlight the interface features and are not part of the actual interface.

Our interactive user interface (Fig. 2) enables users to annotate and track points in video frames, streamlining manual point labeling through multiple annotation layers, and optical flow algorithms for interpolation and refinement.

The interface features three vertically stacked panels: a video display panel for inspecting and annotating points directly on frames, and two trace panels displaying x and y coordinates over time. Annotations appear as scatter points on the video and as traces in coordinate panels, with toggleable dot-like or line-like display styles.

Annotations are organized into layers, each containing all tracked points for a version (e.g., from different annotators or models). Users can visualize two layers simultaneously—a primary layer and a translucent overlay—across all panels. Each point receives a numeric label, and trace panels display the selected point’s trajectory. Users navigate four key elements (video frame, primary layer, overlay layer, and label) via keyboard shortcuts and can jump to specific frames by clicking trace panels.

DUSTrack assists manual annotation through several features: a *guess* function using the Lucas-Kanade algorithm for position estimates, automatic pausing at annotated frames, and a *trim* feature removing incomplete annotations. Users can *toggle* between current and adjacent annotated frames for refining manual annotations.

For model refinement, the UI enables copying annotations between layers, managing them within time ranges, and interpolating values using optical flow. When models fail in specific frames, users can interpolate from surrounding frames, adjust annotations in a new layer, and refine model weights. Annotation layers save to JSON files with frame numbers and 2D coordinates, automatically loading when returning to previously annotated videos for seamless continuation of work across sessions.

DUSTrack’s UI leverages several open-source Python libraries to create an intuitive interface that can be easily customized and extended. The datanavigator library is a wrapper around the popular matplotlib library. It was also created by the authors of DUSTrack and creates the UI and visualizations. Event-driven programming in the datanavigator module enables interactive features like annotation placement and navigation. The pysampled package, also developed by the authors, handles time series data using NumPy and SciPy under the hood. The pandas library primarily ports annotations to and from DeepLabCut. The decord library reads videos, while OpenCV provides the implementation of the Lucas-Kanade optical flow algorithm.

### Datasets

To evaluate DUSTrack and demonstrate its applications, we used three datasets. First, we used an internally collected, publicly available dataset of transverse B-mode ultrasound videos capturing the upper arm during a reaching task in 36 healthy adults^28^. This dataset contains 11 annotated points tracking the triceps, brachialis, and humerus bone to analyze musculoskeletal tissue dynamics.

Second, we assessed DUSTrack’s fascicle tracking capabilities using a public dataset specifically designed for medial gastrocnemius tracking^29^. This fascicle tracking dataset contains data from the gastrocnemius muscle of 5 participants performing plantarflexion movements. This dataset includes annotations of fascicle length and pennation angle from 3 expert raters across 3 separate days—totaling 9 manual annotations for 18 frames per participant. For point tracking, in each video, we annotated 5 points: 2 points on each aponeurosis and 1 point in the muscle belly, and extracted fascicle length and pennation angle from the tracked points.

Finally, we demonstrated cardiac point-tracking applications using five videos from the EchoNet-LVH dataset^30^. Point tracking in this dataset was simpler and less time-consuming than in the other two datasets. Sparse manual annotation combined with LK-RSTC interpolation was sufficient—deep learning was not required. We tracked 4 points in each video to extract relevant cardiac wall motion parameters. These cardiac examples are presented as a practical demonstration of applicability in echocardiography.

### Statistics

Statistical analyses were conducted using the SciPy and statsmodels Python libraries. SciPy was used to perform a one-sampled *t*-test, and paired *t*‑tests for pairwise comparisons and the statsmodels package was used to apply a Bonferroni correction for multiple testing where appropriate. Additionally, SciPy was employed for performing a binomial test to assess whether LK‑RSTC label augmentation yielded superior tracking performance based on a blinded evaluator’s ratings. The reported confidence intervals (CI) are for a confidence level of 95%.

## Results

### State-of-the-art automated point-tracking models demonstrate limited success when applied to ultrasound imaging

Recent advances in automated point-tracking, particularly deep learning-based Track-Any-Point (TAP) methods^31–34^, have achieved impressive performance on natural (RGB) videos. These models enable zero-shot tracking: given a point annotation in a single frame, they track this landmark across the entire video, demonstrating robustness even under prolonged occlusions.

Motivated by the success of TAP models in videos of natural scenes, we investigate whether they can be directly applied to track points in B-mode ultrasound videos. We compare the performance of these zero-shot tracking models with that of a ResNet-50 model fine-tuned for each video. The ground truth was DUSTrack-assisted human annotations that were refined over several (typically 2–4) iterations and manually corrected^28^.

To assess performance and generalization across varied tracking approaches, we survey state-of-the-art TAP models and selected four—CoTracker3^31^, BootsTAP^32^, LocoTrack^33^, and PIPs++^34^. Our selection is based on three key factors: (1) reported performance on TAP-Vid and real-world video datasets, (2) architectural diversity driven by tracking strategies, both context-aware (CoTracker3, which refines tracking using surrounding trackers) and agnostic (BootsTAP, LocoTrack, and PIPs++), and (3) their use of self-supervised learning techniques to improve generalization and reduce the need for annotated data (CoTracker3 and BootsTAP).

**Fig. 3:**
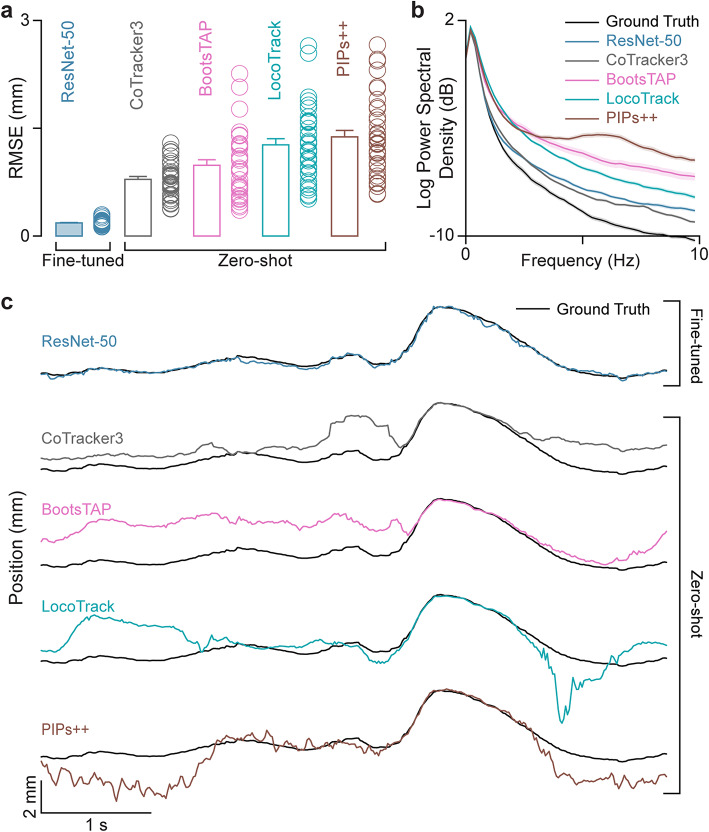
A fine-tuned ResNet-50 tracks points in B-mode ultrasound videos with higher accuracy than current state-of-the-art zero-shot methods. (**a**) Root mean square error (RMSE) in position between the ground truth and the outputs of a fine-tuned ResNet-50 (trained on 25 labeled frames) and four zero-shot tracking models. The ResNet-50 has significantly lower tracking error compared to CoTracker3 (paired *t*-test, *t*_*35*_ = -15.56, *p* = 2.71 × 10^–16^), BootsTAP (paired *t*-test, *t*_*35*_ = -10.68, *p* = 1.49 × 10^–11^), LocoTrack (paired *t*-test, *t*_*35*_ = -12.93, *p* = 6.84 × 10^–14^), and PIPs++ (paired *t*-test, *t*_*35*_ = -13.30, *p* = 3.02 × 10^–14^). Each circle represents the RMSE computed for one video out of 36 total videos. (**b**) Power spectral density of model output for the fine-tuned ResNet-50 and the zero-shot models. All models present higher frequency components compared to the ground truth. (**c**) Representative traces of tracked point position from the fine-tuned ResNet-50 model and zero-shot models relative to ground truth. While the fine-tuned model accurately follows the true point trajectory, the zero-shot models present noticeable tracking errors.

Our results show that ResNet-50 models fine-tuned with 25 human-annotated frames significantly outperform state-of-the-art TAP models in tracking accuracy (Fig. 3a). This demonstrates a clear gap between fine-tuned and zero-shot approaches. Power spectral analysis of point trajectories shows varying levels of high-frequency components, revealing model-specific differences in frame-to-frame jitter (Fig. 3b, c). Notably, CoTracker3 achieves jitter levels closest to the ground truth, even slightly outperforming the supervised ResNet-50 (Fig. 3b, c). This indicates that while CoTracker3 effectively suppresses high-frequency jitter, it remains less precise in localizing the tracked point over time (Fig. 3a-c). Analysis of trajectories over several seconds (Fig. 3c) highlights these distinct differences in jitter levels across models while emphasizing the performance gap between fine-tuned and zero-shot tracking methods, with CoTracker3 emerging as the current leader in zero-shot tracking for ultrasound applications (Fig. 3).

While current state-of-the-art zero-shot tracking solutions have largely mitigated drift, they still need significant improvements to match human-level accuracy and reduce frame-to-frame jitter in ultrasound videos. To address the issue of frame-to-frame jitter, we introduce a filtering method that preserves fast tissue motions while reducing jitter.

### LK-RSTC filtering effectively reduces tracking noise while preserving high-frequency motion

**Fig. 4:**
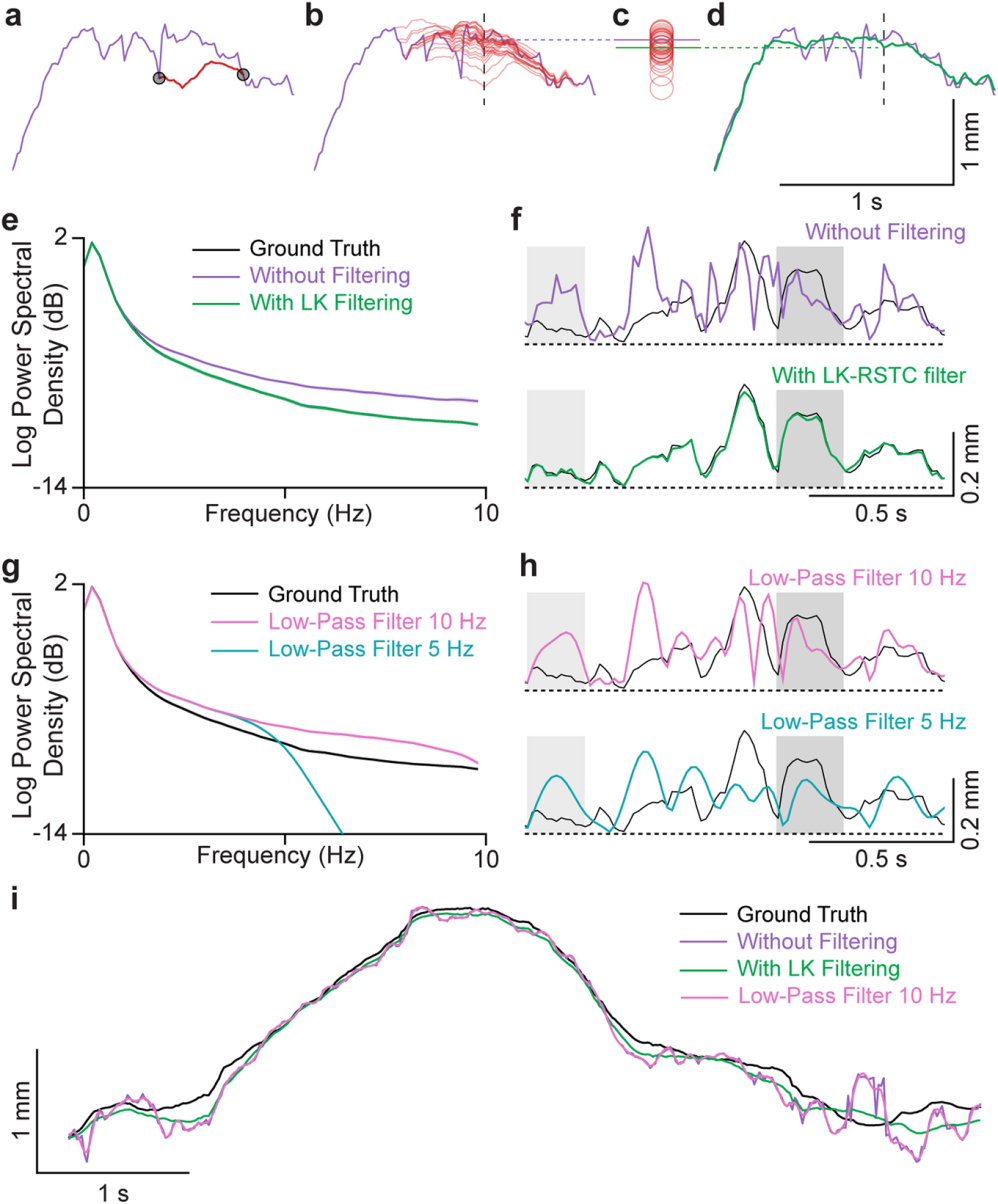
The LK-RSTC postprocessing filter in DUSTrack reduces temporal jitter while better preserving both slow and fast motion dynamics compared to low-pass filtering. (**a**–**d**) Illustration of the LK-RSTC filtering algorithm, applied to the output of a fine-tuned ResNet-50 model. (**a**) Model output (purple) and a tracklet (red) computed using the LK-RSTC algorithm between two points (black circles). (**b**) Visualization of overlapping tracklets (red translucent traces) that contribute to determining the filtered value at the dashed vertical line (red). (**c**) The value of each tracklet at the dashed vertical line in panel b, along with the model output (purple line) and the filtered value (green). (**d**) The original model output (purple) and the result after LK-RSTC filtering (green). (**e**) Power spectral density of the ground truth (black), model output (purple), and LK-RSTC filtered model output (green), averaged across data from 36 participants. The black trace is not visible due to significant overlap with the green trace, and the standard error of the mean (SEM) is too small to be visible. (**f**) Representative point trajectories showing ground truth (black), model output (purple), and LK-RSTC filtered model output (green). The filtered output effectively suppresses high-frequency noise (light gray boxes) while preserving genuine high-frequency motion components (dark gray boxes). All traces are high-pass filtered at 1.5 Hz to highlight differences between filtering methods. (**g**) Power spectral density of the model output with 10 Hz (pink) and 5 Hz (cyan) low-pass filters. The 5 Hz filter excessively attenuates meaningful high-frequency motion, while the 10 Hz filter inadequately suppresses noise in the 2–10 Hz range. (**h**) Representative point trajectories comparing ground truth (black) with 10 Hz (pink) and 5 Hz (cyan) low-pass filtered outputs. The 5 Hz filter suppresses high-frequency tissue motions (dark gray box). (**i**) Representative trajectories of a tracked point comparing LK-RSTC filtering against different low-pass filter settings. Unlike the traces in panels (**f**) and (**h**), these traces are not high-pass filtered. While low-pass filters smooth the signal, they fail to eliminate model errors and suppress genuine motion dynamics. The LK-RSTC filter achieves a better balance, reducing spurious jitter while preserving meaningful high-frequency motion.

The DUSTrack workflow introduces a postprocessing filtering step based on the LK-RSTC algorithm^18^ to leverage the complementary strengths of deep learning and optical flow approaches (Fig. 4a-d). While deep learning models excel at identifying points in individual frames, they often introduce frame-to-frame jitter that can obscure underlying motion patterns. In contrast, optical flow algorithms provide smooth tracking over short durations but accumulate errors over longer periods^18^. Our solution creates multiple short-duration “tracklets” using the LK-RSTC algorithm, with deep learning estimates serving as anchor points (Fig. 4a). By sliding this window frame-by-frame and averaging overlapping tracklet estimates (Fig. 4b, c), we achieve smooth, physically plausible trajectories while maintaining the global accuracy provided by deep learning models (Fig. 4d).

Frequency domain analysis reveals the limitations of traditional low-pass filtering approaches. The LK-RSTC algorithm produces traces and frequency characteristics that closely match the ground truth signal (Fig. 4e, f). While a 10 Hz low-pass filter fails to adequately suppress high-frequency noise, a more aggressive 5 Hz cutoff introduces excessive signal attenuation that distorts the underlying motion patterns (Fig. 4g, h). Representative traces demonstrate this effect qualitatively: low-pass filtered trajectories either show spurious motions or remove faster motion components, whereas the LK-RSTC approach better captures true motion patterns while effectively removing noise (Fig. 4f, h, i).

A more extensive comparison with more complex temporal filters—including Kalman^35^, Rauch-Tung-Striebel^36^, exponential moving average, and Savitzky-Golay^37^ filters—revealed similar limitations (Fig. S1), as these methods, unlike the proposed LK-RSTC filter (Fig. 4), do not incorporate motion information from the video.

### LK-RSTC label augmentation: benefits and limitations

**Fig. 5:**
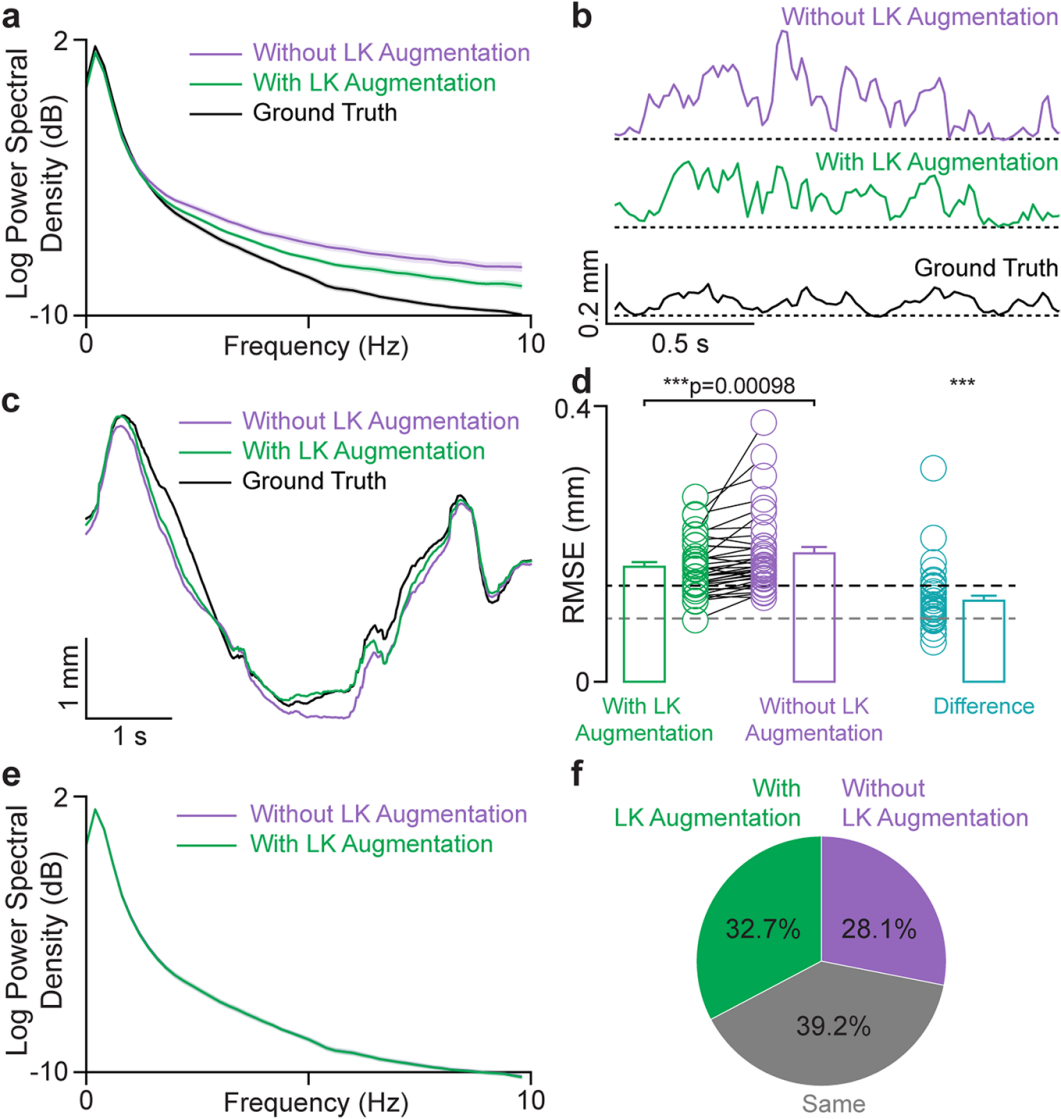
The LK-RSTC label augmentation step in DUSTrack reduces jitter in initial model outputs but this effect is muted after applying an LK-RSTC filter. (**a**) The LK-RSTC label augmentation step occurs prior to fine-tuning a ResNet-50 model. Power spectral density analysis of the ResNet-50 model outputs shows that models trained with LK-RSTC label augmentation produce outputs with reduced high-frequency noise. (**b**) Representative trajectories of a tracked point showing that augmenting training labels with the LK-RSTC algorithm reduces model output jitter. The model outputs are high-pass filtered at 1.5 Hz to highlight the effect of label augmentation on jitter. (**c**) Representative trajectories of a tracked point show that after applying the LK-RSTC filtering step, model outputs exhibit similar temporal jitter and tracking accuracy regardless of whether LK-RSTC label augmentation was used. (**d**) RMSE between the model trained with LK augmentation and ground truth (green), without LK augmentation and ground truth (purple), and between the model with and without LK augmentation (cyan). Error is significantly lower for the model trained with LK augmentation (paired *t*-test, *t*_*35*_ = -3.598, ***p = 0.00098). The difference between the models with LK augmentation vs. without LK augmentation is significantly different from 0 (one-sample *t*-test, *t*_*35*_ = 15.833, ***p = 1.573 × 10^− 17^) after LK-RSTC filtering the outputs of both models. The dashed gray and black lines represent the average differences in tracked point position between the two approaches (with and without LK augmentation). The gray line (92 μm) shows the average difference when the human evaluator could not detect a difference between trajectories, while the black line (140 μm) shows the average difference when differences were detectable. (**e**) Power spectral density analysis confirms that after LK-RSTC filtering, label augmentation has no impact on the frequency content of model outputs. (**f**) In blind scoring by an ultrasound tracking expert, models with and without LK-RSTC label augmentation were perceptually similar after LK-RSTC filtering. The scorer showed no significant preference (binomial test, 127 vs. 109; statistic = 0.538, *p* = 0.268, CI 0.472, 0.603).

LK-RSTC label augmentation is an optional component of DUSTrack for augmenting manual annotations used to fine-tune the ResNet-50 models. To determine its efficacy, we compare the performance of ResNet-50 models with 25 human-annotated frames (across ~500 frames), with and without augmenting the training dataset with predictions in the intermediate frames generated using the LK-RSTC algorithm. Augmenting the training dataset reduces jitter in model outputs (Fig. 5a, b), making model refinement easier.

Although augmentation reduces jitter, upon applying the LK-RSTC filter, the power spectra of trajectories with and without the LK-RSTC label augmentation step are nearly identical (Fig. 5e). However, the results from the two approaches are statistically different (RMSE-wise) from each other (Fig. 5c, d). To better understand the efficacy of the augmentation step, we conduct a systematic evaluation to determine whether any performance differences between the two approaches—with and without augmentation—are perceptible to a human evaluator after applying the LK-RSTC filter. Using 388 point trajectories from 36 videos (one video per participant), we overlay both outputs on each video segment for comparison. A blinded evaluator, unaware of which algorithm produced which output, indicates their preference between the first output, second output, or both. The results do not indicate a systematic preference for LK-RSTC augmentation: 152 trajectories receive no preference, 127 trajectories with LK-RSTC augmentation are preferred, and 109 trajectories without LK-RSTC augmentation are preferred (Fig. 5f). These findings indicate that the impact of LK-RSTC augmentation becomes imperceptible after applying the final LK-RSTC filtering step. Nonetheless, the LK-RSTC label augmentation tool may still serve a valuable role during annotation and model refinement by providing visual feedback during annotation and higher quality tracking during the model refinement stage.

Based on the data from our blind evaluation experiment, we estimate the perceptual threshold at which humans can no longer detect differences between tracked trajectories. To estimate this threshold, we calculated two values: the average difference between outputs when the evaluator could not distinguish between them (90 μm), and the average difference when the evaluator could identify a preferred output (140 μm) (Fig. 5d). We propose that the perceptual threshold for our imaging conditions lies between these two values, at approximately 100 μm. Note that this 100 μm perceptual threshold is not universal—it can vary based on factors beyond imaging parameters, such as display resolution and viewing distance.

### Choosing the number of annotated frames

**Fig. 6:**
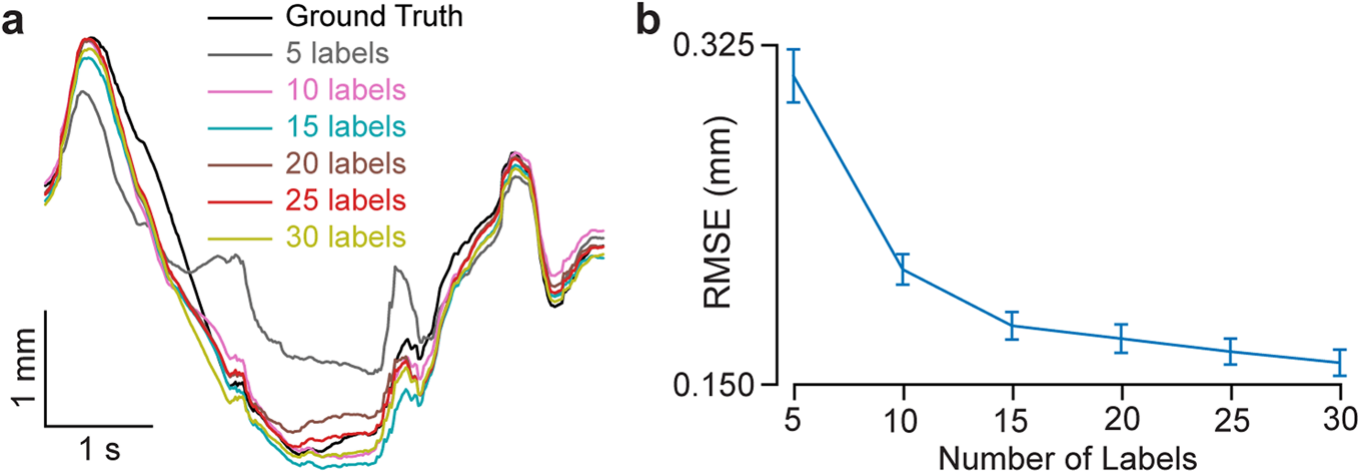
DUSTrack tracking error decreases with the number of labeled frames. (**a**) Representative traces of tracked point position for increasing numbers of labeled frames, from 5 to 30. (**b**) Root mean square error (RMSE) between the ground truth and DUSTrack output as a function of the number of labeled frames. The error decreases substantially between 5 and 15 labeled frames, with only marginal improvement observed between 15 and 30 labeled frames. Error bars indicate SEM across 36 participants.

We then investigate the relationship between tracking accuracy and the number of annotated frames. In videos with cyclic motion patterns, such as cardiac cycles, walking, or reaching, annotating frames within 1–2 motion cycles provides an effective basis for training. This is because human annotators are also prone to introducing drift errors when annotating multiple motion cycles. As expected, increasing the number of manual annotations improves tracking accuracy (Fig. 6). We observe a sharp error reduction when increasing annotations from 5 to 15 frames, followed by diminishing returns beyond this point (Fig. 6a, b). Based on an accuracy-effort trade-off argument, we recommend annotating approximately 25 frames within 1–2 motion cycles for optimal performance.

### Computational performance and workflow timing

We report the approximate time required for each step in the DUSTrack workflow on a computer with a 12th generation Intel i9-12900 CPU and an NVIDIA GeForce RTX 3070 GPU. Initial annotation of 25 labels takes 5–15 min per point, depending on the landmark location and annotator experience—some landmarks are easier to track than others. The optional LK-Augmentation step takes about 2 min for 11 points annotated in 25 frames across a span of 600 frames. Fine-tuning a ResNet-50 model using DeepLabCut v2 (with TensorFlow backend) takes about 4.1 h for 500,000 iterations. DeepLabCut v3 supports the PyTorch backend, which improves training times, and DUSTrack is compatible with both versions. LK-RSTC filtering takes about 10 min with sequential processing for a video of approximately 8,300 frames (a processing speed of ~13 frames/s), and about half that time with parallel processing.

### Clinical and biomechanical applications of DUSTrack

**Fig. 7:**
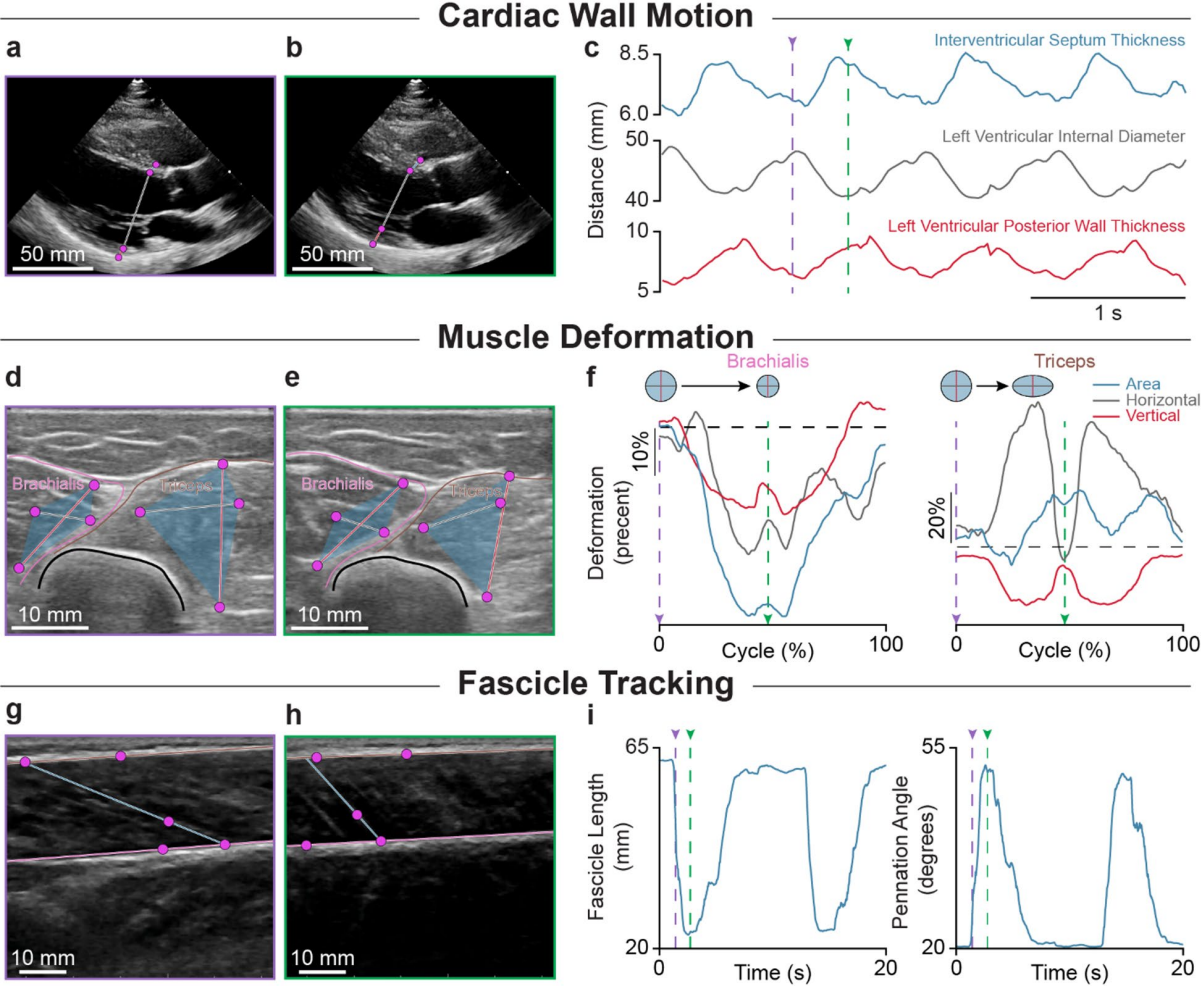
Points tracked via DUSTrack enable extraction of clinically and biomechanically relevant measurements. Applications include cardiac wall motion tracking, muscle deformation tracking, and fascicle tracking. For a video version of this figure, see Supplementary Video 1. (**a**–**c**) Using DUSTrack for cardiac wall motion tracking. Long-axis parasternal echocardiogram frames during systole (**a**) and diastole (**b**), showing tracked points (pink) used to measure cardiac structures: interventricular septum thickness (blue), left ventricular internal diameter (gray), and left ventricular posterior wall thickness (red). (**c**) Time series data showing the measured cardiac parameters across four cardiac cycles. Purple and green dashed lines correspond to the frames shown in (**a**) and (**b**) respectively. (**d**–**f**) Using DUSTrack for muscle deformation tracking. Transverse ultrasound images at the onset of extension (**d**) and retraction (**e**) of the upper arm during a reaching task, showing the brachialis and triceps muscles. Four tracked points per muscle are used to track distances within the muscle cross-section along superior-inferior (vertical, red) and medial-lateral (horizontal, gray) directions. The area enclosed by the four points is shown in blue. (**f**) Muscle deformation patterns during one reaching cycle. The brachialis shows synchronized horizontal and vertical deformations, maintaining shape while reducing area. The triceps exhibits opposing deformations, indicating changing shape, and represented by the circle and oval sketches above. Purple and green dashed lines correspond to the two frames in (**d**) and (**e**) at the onset of extension and retraction, respectively. (**g**–**i**) Using DUSTrack for fascicle tracking. Medial gastrocnemius ultrasound images during the start (**g**) and end (**h**) of ankle plantarflexion. Points tracked on the superficial and deep aponeuroses, combined with fascicle orientation, allow calculation of fascicle length and pennation angle. (**i**) Representative traces of fascicle length and pennation angle, derived from the tracked points. Purple and green dashed lines correspond to the two frames in (**g**) and (**h**), respectively.

Ultrasound imaging is widely used in medical and biomechanical applications to measure important physiological changes, such as the thickness variations of the heart’s interventricular septum during cardiac cycles or muscle fascicle length changes during walking. These valuable measurements can be obtained through point-tracking techniques. To showcase how point tracking can be effectively leveraged to extract these measurements from B-mode ultrasound videos, we apply DUSTrack across three distinct tracking tasks that encompass both clinical and biomechanical applications (Fig. 7).

First, we extract dynamic measurements from parasternal long-axis echocardiograms in the EchoNet-LVH dataset^30^. We measure the interventricular septum (IVS) thickness, left ventricular internal diameter, and left ventricular posterior wall (LVPW) thickness over time. These measurements are essential for assessing cardiac function and diagnosing conditions like left ventricular hypertrophy (LVH), which increases cardiovascular event risk^38,39^. We track points on both sides of the IVS and LVPW using the DUSTrack workflow across four cardiac cycles (Fig. 7a, b, c). For cardiac videos with overlays of tracked points, see Supplementary Videos 1 and 2.

Next, we apply the point-tracking framework to analyze muscle deformations in the upper arm during a reaching task^40^. In a cross-section showing the triceps and brachialis muscles, we track two points in the superior-inferior (vertical) direction and two points in the medial-lateral (horizontal) direction in each muscle to quantify deformations during the reaching movement (Fig. 7d, e). We track these deformations over time (Fig. 7f) by computing them as (D - D₀)/D₀, where D₀ represents the initial distance at the start of the task. This analysis reveals distinct behaviors in both muscles: the brachialis shows correlated horizontal and vertical deformations, indicating changes in cross-sectional area, while the triceps exhibits anti-correlated deformations, reflecting changes in muscle shape.

Finally, we apply the DUSTrack framework to fascicle tracking—a well-studied problem in biomechanics. Modern fascicle tracking algorithms effectively extract fascicle length and pennation angle using classical computer vision algorithms, often achieving excellent results through fully automated processes^20,29,41–45^. In the gastrocnemius muscles, fascicle tracking requires identifying the positions of both aponeuroses and fascicle angles throughout the ultrasound video. We define the upper aponeurosis line by labeling two points, then mark two points on the lower aponeurosis, with one point marking where it intersects a fascicle. We add another point along this fascicle to define the fascicle orientation. Using geometric calculations, we compute the fascicle length (the distance between aponeurosis intersections) and the pennation angle (the angle between the fascicle and lower aponeurosis) (Fig. 7g, h). By calculating these metrics frame by frame, we track changes in fascicle length and pennation angle across multiple cycles of ankle dorsiflexion and plantarflexion (Fig. 7i).

**Fig. 8:**
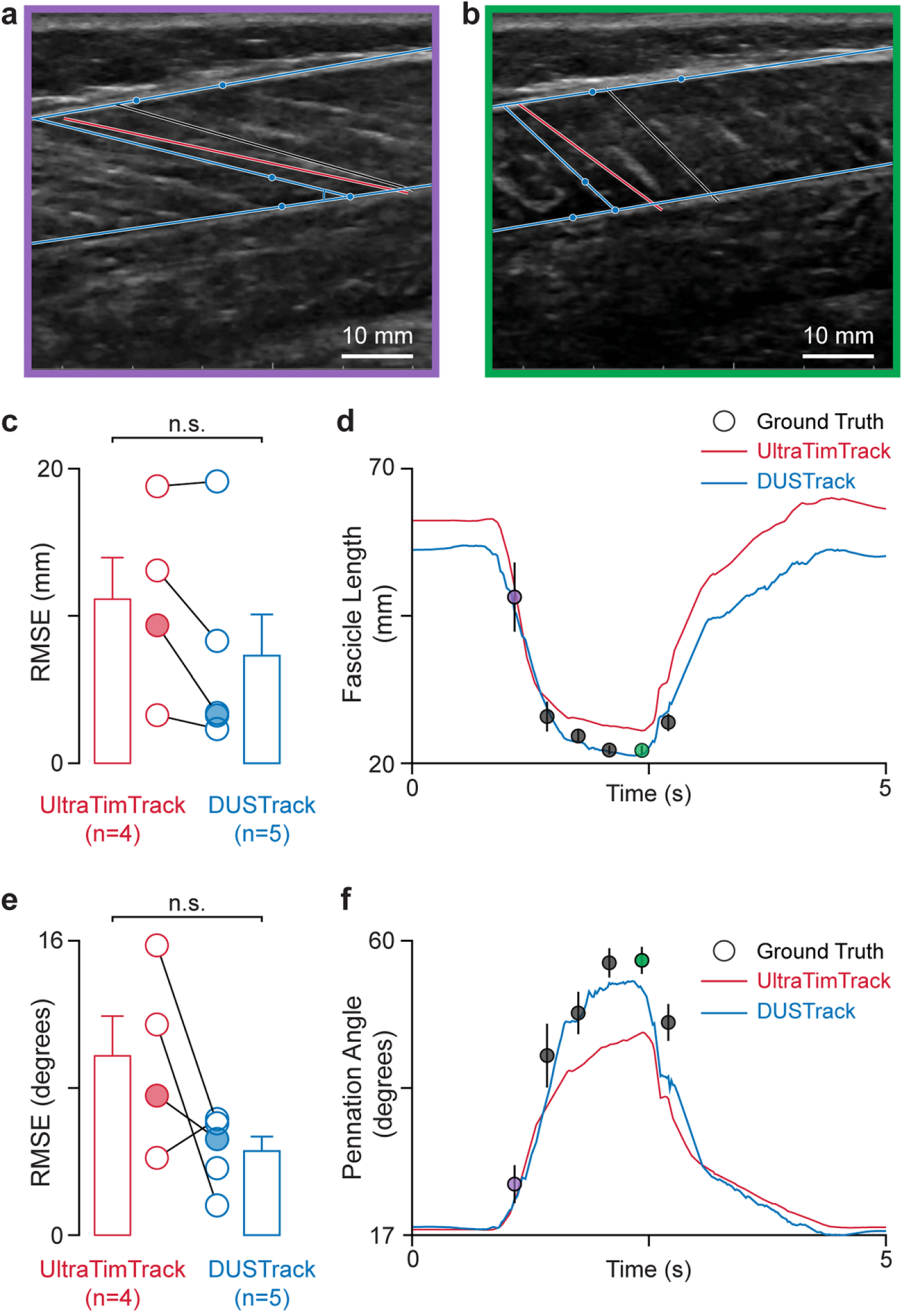
Quantitative comparison of DUSTrack against UltraTimTrack demonstrates comparable accuracy in measuring fascicle length and pennation angle. (**a**,**b**) Ultrasound images of the medial gastrocnemius muscle during ankle plantarflexion show how we measure fascicle length and pennation angle. DUSTrack-tracked points (dots) define the upper and lower aponeuroses and fascicle orientation (blue lines). For comparison, we show UltraTimTrack’s fascicle estimate (red line) and ground truth measurements (gray line). Fascicle length is measured between aponeurosis intersections, while pennation angle is measured between the fascicle and deep aponeurosis. Frames at the beginning (**a**) and end (**b**) of a plantarflexion motion are indicated by purple and green borders. (**c**) Fascicle length measurements show similar accuracy between DUSTrack and UltraTimTrack, with no significant difference in root mean square error (RMSE) (paired *t*-test, *t*_*3*_ = 1.88, *p* = 0.16). (**d**) Representative traces of fascicle length measurements across one plantarflexion cycle show how both methods track changes in length. Black circles indicate ground truth data from manual annotations, with error bars showing SEM across annotations. The purple and green filled circles correspond to the frames shown in (**a**) and (**b**). (**e**) Pennation angle measurements also show comparable accuracy between methods, with DUSTrack showing a trend toward lower RMSE (paired *t*-test, *t*_*3*_ = 1.66, *p* = 0.20). (**f**) Representative traces of pennation angle during one plantarflexion cycle demonstrate how both methods track angle variations. Black circles show ground truth data from manual annotations, with error bars representing SEM across annotations. The purple and green filled circles correspond to the frames shown in (**a**) and (**b**).

We evaluate DUSTrack’s performance against state-of-the-art fascicle tracking algorithms (Fig. 8). For comparison, we use a public dataset^29^ containing recordings from 5 participants—with annotations from 3 expert raters across 3 separate days—totaling 9 manual annotations for each of 18 annotated frames per participant. The average across all 9 ratings is considered ground truth (Fig. 8a, b). We select three existing approaches: UltraTimTrack^20^, HybridTrack^43^, and DL_Track_US^44^ to compare with DUSTrack. The HybridTrack model shows poor performance on this dataset, producing low accuracy and high jitter measurements (Fig. S2). DL_Track_US is unable to identify fascicles in most frames, so no quantitative analysis is possible. Furthermore, prior work has established superior performance of UltraTimTrack relative to HybridTrack and DL_Track_US^20^. Due to these reasons, we focus our quantitative comparisons on UltraTimTrack (Fig. 8). In measuring fascicle length, DUSTrack shows accuracy comparable to UltraTimTrack (Fig. 8c), with DUSTrack slightly underestimating and UltraTimTrack slightly overestimating lengths (Fig. 8d) relative to manual annotation values (ground truth) provided with the dataset. Both methods show comparable accuracy for estimates of pennation angle, with both methods slightly underestimating the angle compared to ground truth (Fig. 8e, f).

Together, these applications highlight the versatility of DUSTrack-based point tracking. In both clinical and biomechanical use cases, DUSTrack can be used to extract various parameters of interest and enable interpretable measurements of diverse tissue dynamics from B-mode ultrasound videos.

## Discussion

### A general-purpose tracking solution for ultrasound

Despite ultrasound’s widespread use in clinical and research settings, most methods that track anatomical landmarks are tailored to specific structures, limiting their adaptability across different regions, orientations, probe configurations, and research applications. In contrast to end-to-end deep learning solutions that directly extract metrics of interest from ultrasound images or videos, such as EchoNet-Dynamic^46^, the ability to track arbitrary points across an ultrasound video enables more flexible and exploratory analyses. Whether monitoring tissue displacement, tracing a vessel wall, examining fascicle orientation, or following a surgical tool, a generalized tracking framework allows researchers and clinicians to extract features most relevant to their application, without the need to develop custom tools for each new task.

One widely used technique for measuring soft tissue deformation is speckle-tracking^47^, particularly in clinical assessments of cardiac function^48^. It employs block-matching or cross-correlation techniques to track the displacement of speckle patterns between frames. However, conventional speckle tracking techniques are application specific and to generalize, typically require tuning with gold-standard reference data obtained through methods such as sonomicrometry, which can be expensive and impractical^49,50^.

In a different domain, fascicle tracking is widely used in biomechanics to measure fascicle length and pennation angle. For example, the architecture of the medial gastrocnemius muscle allows traditional computer vision methods—such as line detection and Hough transforms^20,43^—to fit lines through tracked points along the fascicles and aponeuroses. Yet, these are limited to muscles with specific anatomy where both ends of the fascicle are in view. This limits the number of muscles where fascicle lengths can be tracked using this method.

DUSTrack offers a promising alternative to task-specific tracking methods, serving as a flexible and general-purpose framework for point tracking in ultrasound. It represents a key stepping stone toward the development of zero-shot ultrasound tracking algorithms. As point-tracking methods continue to improve in generalization and accuracy, we anticipate they will ultimately replace conventional tailored algorithms by delivering high-quality measurements with minimal manual intervention.

### Achieving high-quality tracking still requires manual intervention

The ideal point-tracking tool would offer broad generalization, high accuracy, and minimal manual intervention. DUSTrack takes a meaningful step toward this goal by prioritizing generalization and accuracy across diverse ultrasound applications. However, achieving fully automated, zero-shot tracking in ultrasound remains an open challenge.

Foundation models for point tracking are already maturing and will likely yield impressive performance with zero-shot tracking in natural videos. Yet, no general-purpose model currently exists for ultrasound that can match DUSTrack’s accuracy required for clinical and scientific use (Fig. 3). Solutions such as EchoTracker^51^ remain specialized for echocardiograms, while PIPsUS^52^ requires self-supervised training for each new task and falls short of delivering high accuracy tracking.

Reaching this level of automation, while meeting the high accuracy demands of medical imaging, will likely require large-scale, diverse datasets that enable models to generalize across anatomical regions, imaging conditions, and clinical tasks. DUSTrack is well positioned to contribute to this next phase by providing an efficient, user-guided workflow for generating high-quality point-tracking data at scale^28^. Grounded in a data-centric AI perspective^53,54^, DUSTrack prioritizes the accuracy of annotations. Its continued application across varied use cases will be essential for building the training dataset needed to develop future models that move closer to the ideal of zero-shot ultrasound tracking.

Unlike standard computer vision workflows that annotate spatially diverse, disconnected frames, DUSTrack adopts a sequential annotation strategy tailored to the visual complexity of ultrasound imaging. In this context, certain features (such as those within elastic tissues) are more reliably tracked when annotators inspect contiguous frames. This approach improves label consistency, enables effective augmentation through optical flow-based interpolation, and reduces overall manual effort.

A key consideration in evaluating tracking performance is the perceptual threshold—the smallest average displacement a human annotator can detect reliably in tracked trajectories in ultrasound videos. Based on our data collection procedures and the annotators involved, we estimate this threshold to be approximately 100 μm (Fig. 5d). The accuracy of a fine-tuned ResNet-50 model (the first step in our DUSTrack workflow) at approximately 200 μm (Fig. 3a), approaches this threshold, indicating that its performance is nearing the perceptual limits of the human evaluator and the imaging resolution of the dataset (~75 μm per pixel). This threshold also serves as a practical benchmark for evaluating future zero-shot tracking models for B-mode ultrasound.

### A modular framework for customizable ultrasound tracking

Beyond its performance and generalization capabilities, a key strength of DUSTrack lies in its modular architecture. Each component of the framework—annotation, model refinement, and postprocessing—can be used independently, extended for other tasks, or replaced with alternative tools as needed. In the current landscape where this technology is evolving rapidly, DUSTrack’s modular design ensures each component can be independently upgraded as technology advances, providing a flexible foundation for continuous improvement through community contributions.

DUSTrack’s graphical user interface module holds standalone value. Primarily designed for generating accurate ultrasound annotations, the UI prioritizes manual intervention capabilities at every stage of the annotation process. Beyond its primary function, the UI can be used to refine annotations generated by other point-tracking pipelines (requiring minimal code adaptation, easily facilitated through modern LLMs). Its use can extend beyond the ultrasound domain to annotate points in various types of video content where following frames is critical. This adaptability exemplifies the framework’s commitment to flexible components that can be repurposed in other applications.

The deep learning component in DUSTrack can be upgraded as technology evolves. Arguably, instead of ResNet-50^25^ models from DeepLabCut^23,24^, a more recent point-tracking model such as CoTracker3^31^ (the best-performing zero-shot model in our evaluation) would be a more straightforward choice. While CoTracker3 might outperform ResNet-50 on ultrasound videos, it demands substantially greater computational resources, potentially limiting adoption in research settings with hardware constraints. Using ResNet-50 enables point tracking on widely available commercial GPUs with 8 GB of memory. Our approach balances performance needs with practical considerations, making advanced ultrasound tracking technology accessible to a broad range of medical researchers and clinicians.

Another key module of DUSTrack is the LK-RSTC filter, which leverages optical flow to reduce tracking jitter while preserving the true underlying motion captured in the video. Unlike traditional low-pass filters—which may suppress physiologically meaningful high-frequency dynamics—this method uses local frame-to-frame motion estimates to selectively reduce spurious fluctuations without distorting the signal. The result is a smoother trajectory that remains faithful to the original tissue dynamics, contributing to the overall goal of achieving high-accuracy, low-jitter tracking suitable for downstream quantitative analysis and extraction of higher-level metrics.

Although we implemented the LK-RSTC filter using the traditional LK algorithm, it can be replaced with various algorithm variations, such as those using M-estimators^55^ or those incorporating motion sparsity constraints. These alternatives could enhance tracking by improving robustness against imaging artifacts^56^. This flexibility presents opportunities for future toolkit refinements.

### Combining deep learning with optical flow for practical point tracking

The combination of deep learning for spatial feature extraction and optical flow makes point tracking practical and effective. Using LK-RSTC alone requires labeling roughly uniformly spaced frames throughout the video, but this becomes increasingly error-prone as the distance between labeled frames grows, leading to drift accumulation^18^(Fig. S3a). This approach is impractical since human annotations themselves tend to drift over extended time periods. While a ResNet-50-only approach yields usable results, integrating LK-RSTC tools significantly enhances tracking accuracy (Fig. S3b). These observations suggest clear use cases for each approach: LK-RSTC alone works best for short videos of a few hundred frames, while ResNet-50 alone is preferable when out-of-plane motions cause optical flow to infer spurious tissue movements. For most applications, however, the combined pipeline is recommended.

### Broader clinical and scientific impact of general-purpose ultrasound tracking

Our general-purpose ultrasound point-tracking framework can enhance clinical and biomechanical analyses by offering an improved alternative to existing measurement practices. Deep learning tools have advanced cardiac imaging through view classification^57,58^, assessment of cardiac function^46^, and improved predictions of mortality from cardiac data^59^. In this work, we apply similar tools to enhance existing workflows by estimating motion in cardiac data (Fig. 7, Supplementary Videos 1 and 2). Traditional echocardiographic assessments rely on static measurements at end-diastole and end-systole^60^, but DUSTrack can improve this process in two key ways: (1) LK-based tools in DUSTrack’s UI can guide label placement, reducing measurement variability and improving inter-operator consistency; and (2) DUSTrack can enable dynamic analysis throughout the cardiac cycle, capturing time-varying metrics like wall thickness, chamber dimensions, and valve motion. These dynamic measurements provide richer diagnostic insights, particularly for evaluating conditions such as hypertrophic or dilated cardiomyopathy^61,62^ by tracking temporal changes in cardiac structures.

In exercise science, movement analysis primarily focuses on the gross motions of body segments, but the motion of elastic tissues (such as skin, muscle, and internal organs) also carries substantial clinical and scientific relevance. Understanding how these tissues deform and interact during movement provides insights that are less accessible through traditional, joint kinematic analysis alone^40^. Unlike static measurements, which may be sufficient for evaluating certain features like muscle atrophy^63^, dynamic measurements reveal time-dependent behaviors such as strain patterns, tissue velocity, and transient shape changes that could be essential for assessing function, efficiency, and coordination. A persistent challenge in biomechanics has been the lack of non-invasive, dynamic tools to capture these tissue-level motions in vivo. DUSTrack helps address this gap by providing a generalized accessible framework for tracking tissue deformation. The dynamic measurements derived from its point tracking can inform rehabilitation protocols, optimize sports performance, and support injury prevention strategies by offering a more complete view of muscle function during movement^40^.

In addition to the fields of cardiology and biomechanics, we imagine the development of improved ultrasound tracking algorithms will encourage increased adoption of ultrasound by other medical and scientific fields. Since ultrasound provides high frame rate, non-invasive, non-irradiating internal images, we imagine a much broader range of applications is possible once feature extraction is simplified. Some examples include imaging machine parts and developing 3D volume reconstruction algorithms.

### Limitations

Despite the strengths of our methodology, several limitations remain. First, DUSTrack’s accuracy is fundamentally constrained by the quality and consistency of manual annotations. Variability in annotator expertise or intra-session consistency can affect tracking performance. This highlights the need for systematic annotation protocols and potentially for consensus-based labeling strategies to improve standardization.

Second, the performance of DUSTrack’s optical-flow-based refinement depends on video frame rate. Lower temporal resolution can impair its ability to capture rapid physiological motion, diminishing the effectiveness of jitter reduction. Future work should evaluate the toolkit across a wider range of frame rates and imaging conditions to ensure robustness under varied acquisition settings.

Finally, although we demonstrate that DUSTrack generalizes across multiple anatomical regions, its performance in clinical populations remains to be validated. Pathological cases or atypical tissue properties not represented in our dataset may pose additional challenges. Evaluating the toolkit’s robustness in these contexts will be essential for broadening its clinical utility.

## Conclusion

We introduced DUSTrack—a flexible, semi-automated framework for highly accurate point tracking in B-mode ultrasound videos. It combines deep learning with optical flow to address key challenges in ultrasound tracking. Through its modular design, intuitive user interface, and novel filtering techniques, DUSTrack supports high-quality tracking across diverse anatomical structures and motion types. It demonstrates broad applicability and performance comparable to specialized methods, while remaining lightweight and accessible. As an open-source tool, DUSTrack provides a foundation for tissue motion quantification, scalable data generation, and the broader adoption of automated ultrasound analysis in clinical and research settings.

## Supplementary Information

Below is the link to the electronic supplementary material.

Supplementary Material 1

Supplementary Material 2

Supplementary Material 3

## Data Availability

The gastrocnemius dataset used in this study is publicly available from Zenodo, record 2598553 (10.5281/zenodo.2598553). Five videos were used from the EchoNet LVH dataset (10.71718/wtrx-wj56). The upper arm dataset used in this manuscript is available from Figshare (10.6084/m9.figshare.31030252). DUSTrack is publicly available at https://github.com/praneethnamburi/DUSTrack. All other data processing code is available from the corresponding author upon reasonable request.
