# Supplementary material for: DUSTrack: Semi-automated point tracking in ultrasound videos
Source: Sci Rep. 2026 Apr 24;16:13340. doi: 10.1038/s41598-026-42795-3 (PMC13106711; doi:10.1038/s41598-026-42795-3)
Supplement: Supplementary file 1 — Supplementary Material 1 [file 41598_2026_42795_MOESM1_ESM.pdf]

Supplementary Materials for

DUSTrack: Semi-automated point tracking in ultrasound videos

Praneeth Namburi *et al.*

*Corresponding author. Email: praneeth@mit.edu

This PDF file includes:

Figs S1 to S3

Fig. S1. The LK-RSTC postprocessing filter in DUSTrack reduces temporal jitter while better preserving both slow and fast motion dynamics compared to advanced temporal filters.

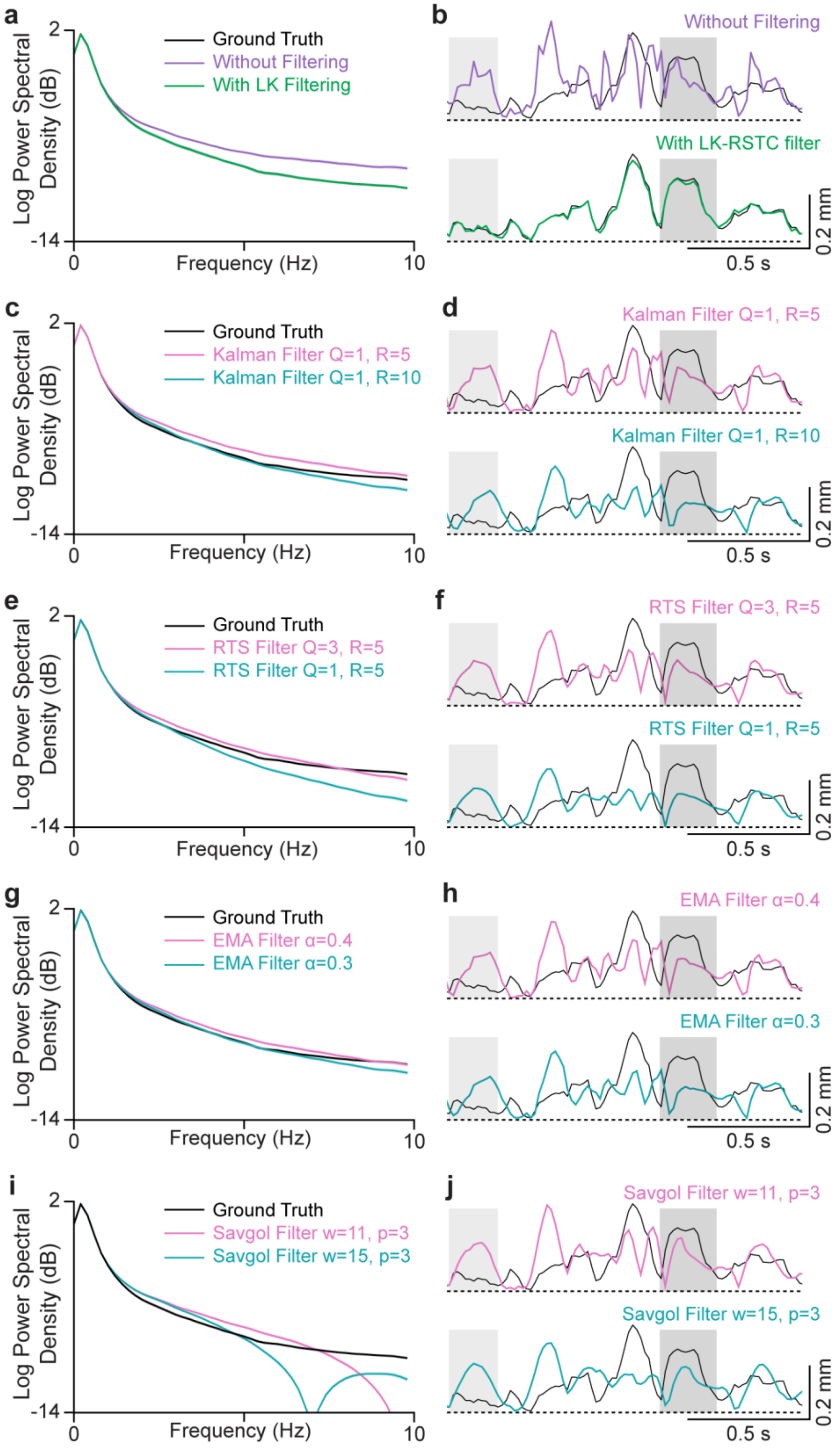

Fig. S1. The LK-RSTC postprocessing filter in DUSTrack reduces temporal jitter while better preserving both slow and fast motion dynamics compared to advanced temporal filters.
(a) Power spectral density of the ground truth (black), model output (purple), and LK-RSTC filtered model output (green), averaged across data from 36 participants. This panel is copied from Fig. 4e to facilitate comparison between our proposed filter and the other filters.
(b) Representative point trajectories showing ground truth (black), model output (purple), and LK-RSTC filtered model output (green). This panel is copied from Fig. 4f to facilitate comparison between our proposed filter and the other filters.
(c-j) Advanced temporal filters applied to the model output, including Kalman, Rauch-Tung-Striebel (RTS), exponential moving average (EMA), and Savitzky–Golay (Savgol) filters. For each method, the left panel shows the power spectral density of the ground truth (black) and two versions of the filter output—a "softer" setting (pink) and a "stronger" setting (cyan)—averaged across 36 participants. The right panel shows representative trajectories with the same color scheme. These filters preserve the overall power spectrum better than a simple low-pass filter but still attenuate meaningful high-frequency motion and fail to fully suppress spurious noise.
(c-d) Kalman filter combining the model output with a constant-velocity dynamical model. A single scalar Q is applied to all state entries and a single scalar R to all measured states; we therefore report only these values. Softer setting: Q = 1, R = 5. Stronger setting: Q = 1, R = 10.
(e-f) RTS smoother obtained by applying a forward–backward Kalman pass with the same constant-velocity. Scalar Q and R values are again applied uniformly across states and measurements. Softer setting: Q = 3, R = 5. Stronger setting: Q = 1, R = 5.
(g-h) EMA filter with different decay factors applied to the model output. Softer setting: $\alpha = 0.4$. Stronger setting: $\alpha = 0.3$.
(i-j) Savitzky–Golay filter applied to the model output. Softer setting: window size w = 11, polynomial order p = 3. Stronger setting: window size w = 15, polynomial order p = 3.

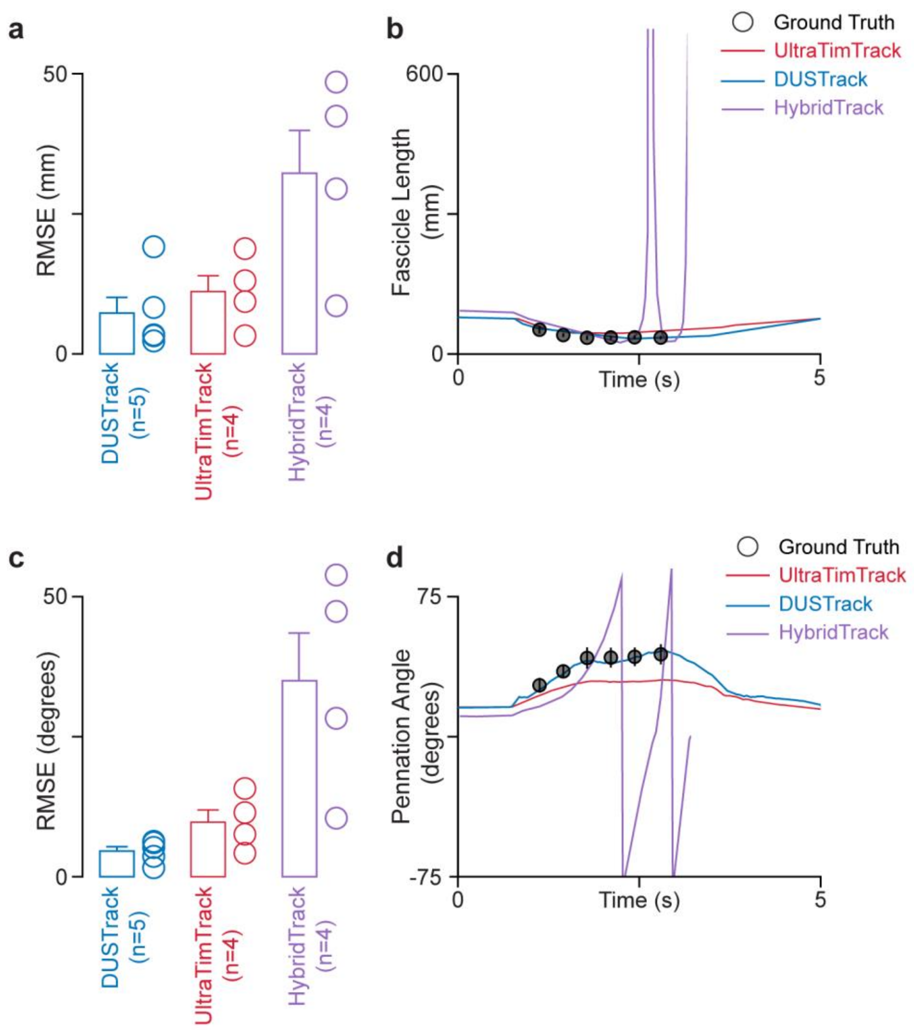

Fig. S2. Fascicle tracking model comparison.
(a) RMSE for fascicle length estimation by DUSTrack, UltraTimTrack, and HybridTrack.
(b) Example traces of fascicle length measurements for each model across one plantarflexion cycle. The black circles represent the ground truth from manual annotations, with the error bars showing SEM across annotations.
(c) RMSE for pennation angle measurements by DUSTrack, UltraTimTrack, and HybridTrack.
(d) Representative traces of pennation angle estimates from each model. Black circles show ground truth data from manual annotations with error bars representing SEM across annotations.

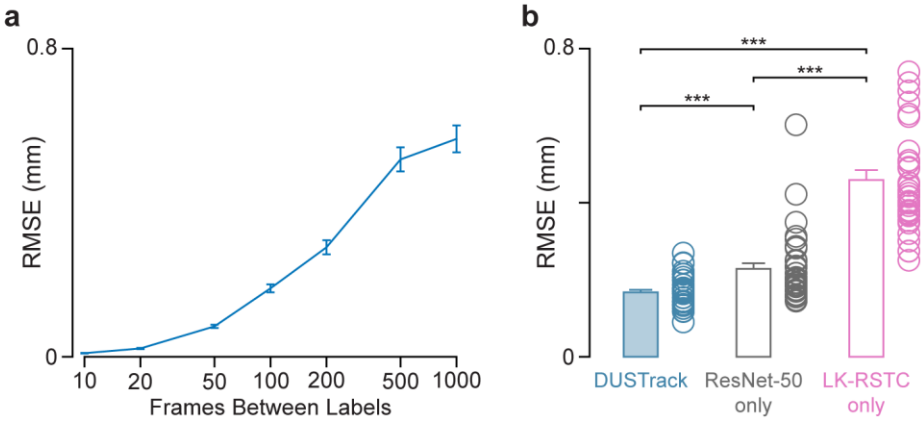

Fig S3. DUSTrack combines a ResNet-50 for feature extraction and the LK-RSTC algorithm to achieve practical and accurate point tracking.
(a) Tracking RMSE for LK-RSTC alone as the number of frames between labeled points increases (x-axis in log scale). As the spacing between labels grows, LK-RSTC increasingly suffers from drift, reflected in rising error.
(b) Tracking RMSE for DUSTrack (ResNet-50 + LK-RSTC), ResNet-50 alone, and LK-RSTC alone when labeling 25 points. DUSTrack has significantly lower tracking error than ResNet-50 alone (paired *t*-test, $t_{35} = -5.72$, $p = 5.44\times10^{-6}$) and LK-RSTC alone (paired *t*-test, $t_{35} = -12.55$, $p = 4.93\times10^{-14}$). ResNet-50 alone also has significantly lower tracking error than LK-RSTC alone (paired *t*-test, $t_{35} = -8.72$, $p = 8.10\times10^{-10}$).
